# Extending the Information Systems Success Model to Explain E-Health Adoption in Saudi Arabia

**DOI:** 10.3390/healthcare14152298

**Published:** 2026-07-29

**Authors:** Homoud Alhaidan, Hussam Al Halbusi, Yaser Hasan Al-Mamary

**Affiliations:** 1 Department of Management and Information Systems, College of Business Administration, University of Ha’il, Hail 81451, Saudi Arabia; 2 College of Commerce and Business (CCB), Lusail University, Jabal Thuaileb, Doha P.O. Box 9717, Qatar; 3 Business Administration Department, College of Business Administration, Al-Bayan University, Airport St, Baghdad P.O. Box Jadirya 2268, Baghdad Governorate, Iraq

**Keywords:** e-health, Information Systems Success Model, technology adoption, patient empowerment, healthcare sustainability, value co-creation, Saudi Arabia

## Abstract

**Highlights:**

**What are the main findings?**

- System quality, information quality, and service quality significantly and positively influence e-health adoption.
- Patient empowerment, healthcare sustainability, and value co-creation generally strengthen the effects of these quality factors on e-health adoption.

**What are the implications of the main findings?**

- Healthcare organizations should focus on improving the quality of e-health systems, information, and services to increase adoption.
- Policymakers and healthcare providers should promote patient empowerment, sustainability, and collaborative care practices to maximize the effectiveness of e-health initiatives.

**Abstract:**

Background/Objectives: Previous studies have found system quality, information quality, and service quality to be significant factors affecting the adoption of e-health services. However, little research has been conducted on factors related to the patient and the healthcare environment that may have an effect on the aforementioned relationship. This paper addresses this gap by extending the Information Systems Success Model to examine the moderating effects of patient empowerment, healthcare sustainability, and value co-creation on the adoption of e-health services in Saudi Arabia. Methods: Data were gathered using purposive sampling through an online survey from 377 adult participants in Saudi Arabia who have previously used e-health systems. Selection of participants with prior experience was done to make sure that their judgments about the quality of the system, information, and the service offered were not made in the absence of usage. Partial Least Squares Structural Equation Modeling was used to examine proposed relationships and moderating effects. Results: System quality (β = 0.312, *p* < 0.001), information quality (β = 0.202, *p* < 0.001), and service quality (β = 0.274, *p* < 0.001) significantly influenced the adoption of e-health services. Patient empowerment and healthcare sustainability significantly strengthened the relationships between all three quality dimensions and e-health adoption. Value co-creation had a significant effect on the relationships between system quality and e-health adoption, as well as between service quality and e-health adoption. Nonetheless, value co-creation failed to have any significant moderating effect on the relationship between information quality and e-health adoption (β = 0.106, *p* = 0.126). This shows that its moderating influence was not consistent across all quality dimensions. Conclusions: The findings demonstrate that system quality, information quality, and service quality are important determinants of e-health adoption. In addition, patient empowerment, healthcare sustainability, and value co-creation serve as important contextual factors that enhance the effects of quality dimensions on e-health adoption, although variations exist across specific interactions. These findings show that patient and healthcare conditions need to be considered when discussing the impact of system, information, and service quality on adoption of e-health, while at the same time providing context-based insights for the healthcare sector in Saudi Arabia.

## 1. Introduction

Despite the increased investments in the provision of digital health services, there is still inconsistency in terms of patient uptake, suggesting that the presence of e-health technologies does not necessarily translate to their use. In previous research, adoption has been found to be a function of the dependability and usability of digital technologies, the validity and usefulness of health information, and the level of support that users receive. Nonetheless, a lot of research in this area has focused on analyzing these quality elements as predictors of adoption without considering factors such as the patient and healthcare context that could affect the effect of the predictors. This is an important gap because the way patients adopt the same technology could differ depending on their ability to be involved in the process of care, the supportive nature of the healthcare context, and the opportunities for collaboration with healthcare providers.

According to the Information Systems Success Model, the technology conditions related to the adoption of e-health systems are categorized under system quality, information quality, and service quality [1]. Within an e-health context, system quality pertains to the accessibility, reliability, usability, security, interoperability, and scalability of the technological platform [2,3]. On the other hand, information quality pertains to the correctness, completeness, relevance, and timeliness of the health information made available through the system, while service quality refers to the responsiveness, availability, and effectiveness of the service given by the system. These dimensions give a logical foundation for understanding how patients assess e-health services and their willingness to adopt such services. Thus, this research paper concentrates on system quality, information quality, and service quality as key quality factors for e-health adoption.

Previous studies that have applied the Information Systems Success Model tended to consider the impact of system quality, information quality, and service quality on technology use and adoption directly [4,5]. While this method identifies whether quality is an influencing factor on technology adoption, it offers fewer insights into the specific conditions under which patients have the capability and willingness to adopt e-health based on their quality assessments. This is due to the heterogeneity among patients in terms of their health literacy, decision-making power, and involvement in the processes of healthcare delivery [6,7,8]. Healthcare environments may differ in their capacity to provide patients with accessible, efficient, and reliable digital services over time, and this should also be taken into account [9,10].

The current study explores the role of three additional contextual factors: patient empowerment, healthcare sustainability, and value co-creation. Patient empowerment can be defined as the knowledge, confidence, and control possessed by patients for making use of the benefits provided by e-health systems [6,11]. Healthcare sustainability is understood as the ability of the healthcare environment to provide patients with accessible, resource-efficient digital healthcare services over the long term [9,12]. Value co-creation is the joint efforts made by patients and healthcare providers for generating healthcare experiences and outcomes [13,14,15]. These factors are explored as moderators, as they are expected to influence the relationship between system quality, information quality, and service quality, on the one hand, and e-health adoption, on the other.

Saudi Arabia provides an appropriate setting for conducting this study since there is currently a massive digital transformation of the Saudi healthcare sector within the framework of the Vision 2030 reform. This program places special emphasis on the development of technology-enabled healthcare, increased accessibility, efficiency, and the introduction of patient-centered care [16]. Nevertheless, investment in technology and policies cannot guarantee the adoption of e-healthcare services by patients. Patients’ perception of the reliability of the system, information, and support during usage plays a great role in this process [17]. Thus, the Saudi Arabian setting is useful for researching whether patient empowerment, healthcare sustainability, and value co-creation impact the degree to which those quality dimensions lead to the adoption of e-health services. Nevertheless, the results of the research should be considered as context-specific.

The study offers three distinct contributions. First, it contributes to the Information Systems Success Model by going beyond the direct influence of system quality, information quality, and service quality to study the circumstances under which these components impact e-health adoption in a stronger or weaker way. Second, the study offers patient-level insights about the moderating effects of patient empowerment, sustainability of healthcare services, and value co-creation—constructs that have not been given much attention in the application of the Information Systems Success Model to the field of digital healthcare. Third, by exploring these influences in Saudi Arabia, the study provides context-specific insights from a healthcare system that is rapidly evolving digitally.

To operationalize these contributions, the research will have two main objectives. First, it will assess the effect of system quality, information quality, and service quality on the use of e-health services. Second, it will analyze the role of patient empowerment, healthcare sustainability, and value co-creation in mediating these relationships. Accordingly, the study addresses the following research questions:

1. How do system, information, and service quality influence patients’ adoption of e-health services?
2. How do patient empowerment, healthcare sustainability, and value co-creation moderate these relationships?

## 2. Theoretical Background

The Information Systems Success Model was developed to show that the quality of information systems impacts their use, user satisfaction, and perceived benefits. The original model focused on the quality of an information system and the quality of information provided by it as the two key factors of information system success, while the updated version included service quality and explained the relationships between intention to use, actual use, user satisfaction, and net benefits [1]. This model is relevant to e-health since it differentiates the technical performance of the digital platform from the quality of information it provides and the quality of service offered to the users.

The model was applied to hospital information systems, electronic health records, and online health information services. According to current empirical evidence, system quality, information quality, and service quality play significant roles in the process of system usage and user satisfaction and the perception of their benefits regardless of the particular technology used in healthcare and the type of users, but the magnitude and significance of these relationships vary [18,19,20]. For instance, according to Bashiri et al. [21], the quality of the system was the most crucial dimension in evaluating a national electronic health record system, yet the users had low perceptions of the benefits of the system. In turn, Ikenyei and Haggerty [22] proved almost all relationships suggested by the model within the public health information system context. However, they showed that information quality does not predict system usage. Thus, the findings demonstrate that favorable perceptions of quality lead to different results in different healthcare environments.

Although the model has been used frequently for analysis of the healthcare environment, most studies based on it examined only direct relationships between the variables mentioned above. Moreover, much of the evidence collected using this model was obtained from healthcare professionals and organizational information systems. Therefore, the model provides strong evidence that quality matters in healthcare information systems, but does not explain when and how these perceptions result in the adoption of systems. The present study tries to resolve this issue by testing whether patient empowerment, healthcare sustainability, and value co-creation moderate relationships between the three quality dimensions and e-health adoption.

Patient empowerment reflects the extent to which individuals are informed, engaged, and capable of making decisions regarding their health. Healthcare sustainability refers to the long-term viability and efficiency of healthcare systems in delivering consistent and equitable services. Value co-creation captures the collaborative process through which patients and healthcare providers jointly generate value during service interactions.

These moderators are theoretically important because they function as boundary conditions. For instance, even when system, information, and service quality are high, their impact on adoption may vary depending on the level of patient empowerment, the sustainability of the healthcare system, and the extent of collaborative value creation between stakeholders.

System quality entails the technical capabilities of the information system under consideration. For the patient, system quality in the case of e-health includes reliability, accessibility, flexibility, response time, usability, compatibility, and security of the platform. These qualities impact the effort and uncertainty required for the use of the service. An unreliable and non-accessible platform that is hard to navigate or responds slowly may reduce the willingness to use the platform regardless of the value of the service offered by the provider of healthcare. On the other hand, a platform that is reliable and accessible will allow a person to perform healthcare activities in a more efficient way. Assessments of the e-health system have also revealed that operational reliability, response time, access procedures, and system integration were critical factors of users’ perception of the success of the system [1,18,21].

**H1.**  *System quality positively influences the adoption of e-health services.*

Information quality entails the positive attributes of information created, processed, and transmitted by means of an information system. In e-health services, it involves the accuracy, completeness, relevance, timeliness, clarity, and consistency of the health information offered to patients. These attributes are especially important in healthcare as patients use digital information when evaluating their health status, before a consultation, adhering to treatment advice, or making health care-related decisions. The provision of inaccurate, incomplete, or obsolete information will lead to ambiguity and reduce trust in the service, while the delivery of clear, relevant, and up-to-date information increases understanding and enhances the perceived usefulness of the platform. Previous studies employing the Information Systems Success Model in the context of online health information services and electronic health records have regarded information quality as one of the core criteria of user evaluation of digital healthcare services [1,20,21]. Thus, patients are more likely to use e-health services when the information is evaluated as accurate, useful, and relevant to their health care needs.

**H2.**  *Information quality positively influences the adoption of e-health services.*

Service quality refers to the level of user assistance and support during a user’s engagement with an information system. In e-health, it relates to the responsiveness of medical and technical professionals, their availability for support, the quality of communication, problem-solving abilities, and assurance that is provided to patients in terms of digital service delivery. Users assess not only the platform and its content but also the kind of help they are offered when problems occur. Quick help and support are necessary for reducing patients’ uncertainty and increasing their trust in digital healthcare. However, failure to communicate or solve problems on time may prevent patients from using the platform regardless of the fact that it operates effectively. The notion of service quality differs conceptually from system quality since the former relates to human and organizational assistance in terms of digital platforms while the latter refers to the technological quality of the platform itself [1,20]. Therefore, patients’ perception of service quality affects the adoption of e-health services.

**H3.**  *Service quality positively influences the adoption of e-health services.*

While there is some level of relationship between the concepts of system quality, information quality, and service quality as factors of success of information systems, these three constructs are separate dimensions of user assessment of an e-health care facility. The first relates to the technical and functional characteristics of the system; the second refers to the nature of the health care information delivered through it; while the third pertains to the human and organizational services associated with the use of the platform [1]. They should therefore be modeled as separate predictors in order to estimate their separate impacts on the adoption of e-health facilities.

While patient empowerment, healthcare sustainability, and value co-creation might co-exist in digital healthcare, each concept represents a separate element of the adoption environment, and thus it would be inappropriate to use them interchangeably. Patient empowerment refers to an individual-level capability that pertains to the patient’s level of knowledge, confidence, autonomy, and ability to make decisions in relation to healthcare [6,7]. Healthcare sustainability is a system-level attribute that reflects the capacity of healthcare services to be sustainable in terms of accessibility, efficiency, equity, and reliability [9]. Value co-creation refers to a relationship between the patient and healthcare providers who create value for patients together by sharing knowledge, exerting efforts, making decisions, and giving feedback [13,14]. Therefore, patient empowerment relates to what the patient can do, healthcare sustainability relates to what the healthcare system can maintain, and value co-creation relates to what patients and providers do together.

These contextual factors should not be assumed to necessarily foster the uptake of e-health in all situations. Information access and participation in care may help ensure that patients are better informed about their condition; however, too much information, overly complex or not properly explained information, may cause confusion or cognitive burden or may even increase anxiety in patients receiving such sensitive information without timely explanation by healthcare professionals. As shown in the literature on patient portals, for example, there are some concerns among both patients and healthcare professionals regarding the potential risk of overwhelming users with medical information or increasing anxiety [23,24], whereas more recent research highlights how access to test results pending on the portal may prompt patients to frequently check the portal and communicate with healthcare professionals. Likewise, patient empowerment and value co-creation may not prove particularly useful if patients lack adequate digital or health literacy, do not want to assume an active role in their care process, or are not provided with responsive healthcare professionals.

### 2.1. Patient Empowerment as a Moderator

Patient empowerment refers to the ability of people to have an understanding of health-related information, control over the healthcare processes and management, and participation in healthcare [6,11]. Within the sphere of digital healthcare, empowered patients would be able to comprehend information provided, communicate their needs, evaluate the treatment options, and feel confident about using various e-health tools. The provision of patients with access to health records, remote communication with health professionals, and real-time monitoring could help increase patients’ participation in their health and make decisions based on the provided information [25,26]. As a result, empowered patients could recognize and utilize all benefits of high-quality e-health systems.

Patient empowerment could change the strength of the relationship between quality and the adoption of e-health systems. When the quality of the system is high, it means that empowered patients could be more likely to benefit from all reliable, accessible, and useful functions provided, since they are going to have the necessary level of confidence and skills. Regarding information quality, empowered patients may be able to use the information provided and apply it to their decisions due to high levels of health literacy and decision-making ability [8]. Moreover, patient empowerment could also increase the impact of service quality, since empowered patients are able to communicate their needs and participate in their care process actively [27].

**H4a.**  *Patient empowerment moderates the relationship between system quality and the adoption of e-health services, such that the relationship is stronger at higher levels of patient empowerment.*

**H4b.**  *Patient empowerment moderates the relationship between information quality and the adoption of e-health services, such that the relationship is stronger at higher levels of patient empowerment.*

**H4c.**  *Patient empowerment moderates the relationship between service quality and the adoption of e-health services, such that the relationship is stronger at higher levels of patient empowerment.*

### 2.2. Healthcare Sustainability as a Moderator

Within the scope of this study, healthcare sustainability is defined by the ability of digital healthcare services to remain accessible, efficient, reliable, and adequately supported. In other words, it implies the continuity of delivery of digital healthcare services, the support from institutions, resource availability, and the possibility for patients to access digital healthcare services whenever needed [9,10]. The construct is not used as a more general term related to environmental sustainability in the absence of explicit representation of environmental outcome indicators within the measurement items. Such an interpretation of the concept aligns with the focus on patients within the framework of this research, as it reflects patients’ perception of the reliability of digital healthcare services within the healthcare system.

Healthcare sustainability is used as a moderator variable since it might impact the conversion of favorable perception of the quality of e-health services into their adoption. In the case of the stability and adequate support of digital healthcare services, patients will benefit from the advantages of reliable systems, accurate information, and responsive support more often. Thus, regarding the stability of e-health services, system quality, information quality, and service quality will significantly impact their adoption. On the contrary, in the case of lack of regularity in access, instability of infrastructure, or insufficient institutional support, the positive assessments of quality will influence the adoption of the technology less strongly.

**H5a.**  *Healthcare sustainability moderates the relationship between system quality and the adoption of e-health services, such that the relationship is stronger at higher levels of healthcare sustainability.*

**H5b.**  *Healthcare sustainability moderates the relationship between information quality and the adoption of e-health services, such that the relationship is stronger at higher levels of healthcare sustainability.*

**H5c.**  *Healthcare sustainability moderates the relationship between service quality and the adoption of e-health services, such that the relationship is stronger at higher levels of healthcare sustainability.*

### 2.3. Value Co-Creation as a Moderator

Value co-creation is defined as a collaborative process where patients and health professionals work together and combine efforts to deliver healthcare [13,14,28]. Simply put, it means that both parties participate in the process. It should be differentiated from patient empowerment, which involves knowledge, skills, autonomy, and confidence of individual patients. Patient empowerment might help individuals participate, but value co-creation is achieved only when patients and healthcare providers interact to develop a healthcare experience.

Digital health innovations offer possibilities for value co-creation because they provide opportunities for patients to use information about health, express preferences, give feedback, and take part in decision-making regarding their care [29,30,31]. Through such interaction, healthcare providers can have more understanding of the needs of patients and adapt digital services based on this. In this way, higher levels of participation can result in higher relevance of the service, more patient engagement, and higher value perception of e-health solutions [15,32].

Value co-creation may reinforce the impact of each quality dimension on the adoption of e-health systems. Reliability and usability of e-health solutions represent the technical prerequisites for effective interaction between patients and health professionals. Relevant and accurate information offers a common ground for dialog and shared decision-making. Responsiveness and effective communication can also facilitate the participation of patients in the process of care delivery. Therefore, when the level of value co-creation is high, patients can convert positive perceptions of the quality of the system, information, and service into actual adoption of e-health solutions.

**H6a.**  *Value co-creation moderates the relationship between system quality and the adoption of e-health services, such that the relationship is stronger at higher levels of value co-creation.*

**H6b.**  *Value co-creation moderates the relationship between information quality and the adoption of e-health services, such that the relationship is stronger at higher levels of value co-creation.*

**H6c.**  *Value co-creation moderates the relationship between service quality and the adoption of e-health services, such that the relationship is stronger at higher levels of value co-creation.*

**Figure 1:**
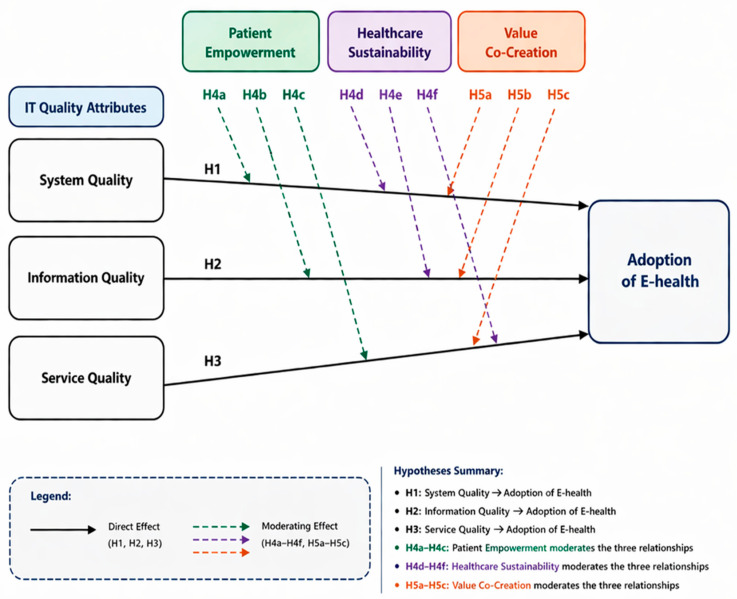
Research model.

Figure 1 depicts the conceptual framework for this study. E-health adoption is modeled to be affected independently by system quality, information quality, and service quality. Patient empowerment, healthcare sustainability, and value co-creation are also modeled to be independent moderators. Patient empowerment is defined as the ability of patients to comprehend and benefit from the services provided by e-health technology. Healthcare sustainability is defined as the ability of the healthcare context to ensure accessible and dependable digital services for healthcare. Value co-creation is defined as the process of collaboration in which patients and healthcare practitioners jointly provide healthcare services.

In this model, the relationship between system quality, information quality, service quality, and e-health adoption is moderated by patient empowerment, healthcare sustainability, and value co-creation. Direct relationships are separated from moderating relationships in order to distinguish established relationships from moderating factors. The three quality dimensions have been defined separately but related to one another in terms of predictors, as the model intends to measure the individual impact of these dimensions on the adoption of e-health.

## 3. Method

### 3.1. Sample and Process

The study participants were selected by a purposive sampling technique involving adult patients who had experience using e-health services before. Invitations to fill out the Google Forms survey were sent to 500 people via WhatsApp and Facebook groups. Before proceeding to complete the questionnaire, prospective participants had to confirm their eligibility for the study by answering the screening question asking whether they have used an e-health service before, such as telemedicine or electronic health records. Individuals who answered negatively to having ever used an e-health service were not eligible to complete the questionnaire. Out of 500 survey invitations, 423 questionnaires were returned for a response rate of 84.6%. After the screening procedure, 46 questionnaires were excluded from analysis due to incompleteness, duplication, or failure to meet the eligibility criteria. As a result, the study sample included 377 valid responses. The choice of the tool was due to its user-friendliness and anonymity.

The sufficiency of the obtained sample was tested using G Power 3.1 software. Sample size estimation was done using the linear multiple regression model. The number of predictors was equal to 15, f^2^ was assumed to be 0.15, alpha value was 0.05, and statistical power was 0.95. As a result, the minimum required sample size was 199 participants. The obtained sample of 377 responses met the requirements and was sufficiently large.

### 3.2. Ethical Considerations and Informed Consent

Ethical clearance for this study was granted by the Research Ethics Committee of the University of Ha’il on 19 January 2026, under reference number H-2026-015. Ethical clearance was sought prior to the commencement of data collection in February 2026. Participation in the study was voluntary, and electronic informed consent was obtained prior to giving the participants access to the questionnaire. The consent page gave reasons for conducting the study, described the process involved, indicated the purpose of the data collection, outlined the voluntary nature of participation, and stated that the participants had the right to terminate their participation in the survey at any time without penalty. Contact information of the researchers was included in case the participant wished to ask questions before giving consent. No personal information was collected, and answers were analyzed anonymously. The participants had the option of withdrawing from the survey before submission by simply closing the survey without submitting the answers. Since responses were anonymous and could not be traced back to an individual, the answers could not be traced and withdrawn upon submission.

### 3.3. Variables Measurement

Given that the participants are Arabic speakers, Brislin’s [33] back-translation approach was employed to convert the English version into Arabic. This approach is frequently employed in cross-cultural surveys to assess the accuracy of translations. Initially, the items were translated into Arabic and then compared to the original English version by two bilingual individuals to detect semantic disparities. Where inconsistencies were identified, the translation process was repeated until equivalence between both versions was achieved. In the back-translation phase, the Arabic version of the questionnaire was analyzed by 2 experts in health informatics and information systems. The analysis was conducted regarding the clarity, pertinence, linguistic adequacy, and cultural appropriateness of the items for adult users of e-health in Saudi Arabia. The linguistic equivalence of the questionnaire was also reviewed by an Arabic–English bilingual language specialist. The expert compared the English and Arabic versions of the questionnaire and suggested modifications if the expressions used were found vague or were not suitable for the Saudi healthcare environment. This analysis was done before the administration of the questionnaire. Responses were measured using a five-point Likert scale ranging from 1 = Strongly Disagree to 5 = Strongly Agree. All measurement items were adapted from previously validated scales to ensure reliability and construct validity.

Information quality was measured using four items adapted from DeLone and McLean [1] and Lin [34]. The items assessed respondents’ perceptions of the accuracy, completeness, relevance, and timeliness of information provided through e-health services. System quality was measured using nine items adapted from the same sources, covering the reliability, accessibility, flexibility, usability, and functional performance of the digital platform. Service quality was assessed using five items adapted from DeLone and McLean [1] and Lin [34]. Patient empowerment is an example of a reflective formative higher-order construct. The items were considered as reflective measures for their corresponding first-order constructs, which include health literacy, patient participation, patient control, and communication with health care professionals. This is because the dimensions listed above are different components of patient empowerment that are not substitutable with each other. A patient, for instance, can have high health literacy but lack the ability to control health care-related issues or communicate well with health care professionals. It would change the meaning of the construct if one of these dimensions were taken out. This follows from the difference made in the literature on PLS-SEM regarding the two types of higher-order constructs [35,36]. Healthcare sustainability was evaluated through several indicators that had been created for the purposes of this study based on the existing literature on accessible, efficient, reliable, and sustainable provision of digital healthcare. The indicators reflected participants’ perception of e-healthcare as being consistently available, sufficiently supported, resource efficient, and maintainable. Content validity was determined by specialists in the fields of health informatics, information systems, and healthcare management in terms of relevance, clarity, and comprehensiveness of the indicators. Changes were made according to their recommendations prior to the questionnaire administration. Value co-creation was assessed using two dimensions: patient participation behavior (10 items) and patient citizenship behavior (5 items), adapted from Russo et al. [37]. Value co-creation was operationalized as a reflective formative higher-order construct. The constituent items were considered reflective measures of patient participation and citizenship behavior, while the two dimensions were considered formative at the higher-order level. Patient participation behavior entails the behaviors necessary for service provision, which include information exchange, responsibility, interpersonal communication, and cooperation. On the other hand, patient citizenship behavior entails the extra behaviors performed by the patient in addition to fulfilling his primary duties, such as feedback, advocacy, helping other patients, and tolerance. These two constructs are quite different from each other and cannot be used interchangeably. A patient can be an active participant in health care delivery yet not perform any voluntary behaviors of citizenship. Finally, the adoption of e-health was measured using eight items adapted from Wu et al. [38]. The items assessed respondents’ intention to use, continue using, and rely on e-health services in future healthcare activities. The construct therefore represents behavioral intention towards e-health adoption rather than verified usage behavior.

## 4. Data Aggregation and Results

To validate the proposed relationships, this study utilized SmartPLS 4 software to perform Partial Least Squares Structural Equation Modelling (PLS-SEM) [39]. PLS-SEM was selected due to its suitability for analyzing complex models involving multiple constructs and moderating effects, as well as its ability to handle relatively non-normal data distributions and moderate sample sizes [40].

This approach is particularly appropriate for predictive and exploratory research where the objective is to examine relationships between latent constructs rather than confirm a well-established theory. A bootstrapping procedure with 5000 resamples was conducted to estimate the significance of path coefficients. This procedure enables the calculation of standard errors, t-values, and *p*-values for hypothesis testing.

### 4.1. Common Method Variance (Cmv)

Since all the constructs were assessed through the use of a single instrument where participants responded to it, certain procedural steps have been applied within the questionnaire in order to mitigate common method variance. Participants were told that their responses would be kept anonymous and that there are no right or wrong answers in order to minimize evaluation apprehension and socially desirable responding. The wording of the questionnaire is clear and concise; at the same time, questions on the predictor, moderator, and outcome constructs were placed in different sections of the questionnaire. Question order was also structured in such a way as to minimize the ability of participants to understand the predicted relationship between the constructs.

Statistical evaluation of common method variance was done as well. In performing Harman’s single-factor test, it was found that the first factor that was not rotated contributed to 13.82% of the total variance, which is less than the typical cut-off point of 50%. Furthermore, an analysis of full collinearity was done based on variance inflation factor values. As can be seen in Table 1, the values ranged between 1.137 and 2.101 and were below the cut-off point of 3.3, according to Kock [41].

Nevertheless, these diagnostic procedures cannot completely exclude common method variance, and the results should be interpreted with this limitation in mind.

### 4.2. Measurement Model Assessment

When analyzing a latent construct, it is crucial to make a well-considered decision on whether to use reflective or formative indicators [35,36]. Reflective measurement is appropriate when indicators are interchangeable and are assumed to be caused by the underlying construct. In contrast, formative measurement is used when indicators collectively form the construct and are not necessarily interchangeable [36,42]. To ensure appropriate model specification, the distinction between reflective and formative constructs was guided by criteria such as direction of causality, indicator interchangeability, covariation, and whether indicators represent antecedents or consequences of the construct [36].

Regarding reflective constructs, indicator reliability was assessed using outer loadings. The results show that all item loadings exceeded the recommended threshold of 0.707 [43], as presented in Table 2. Internal consistency reliability was evaluated using composite reliability (CR), with values ranging from 0.744 to 0.871, all exceeding the recommended threshold of 0.70. Convergent validity was assessed using the average variance extracted (AVE), with values ranging from 0.571 to 0.715, all above the minimum threshold of 0.50 [43]. These results confirm that the reflective measurement model demonstrates satisfactory reliability and convergent validity.

The higher-order formative constructs were evaluated through the existence of collinearity between the dimensions of the constructs and the significance and validity of the outer weights. The variance inflation factor was below the suggested value of 5, which suggests that there was no issue of collinearity in the dimensions of the formative constructs [43].

Significance of the outer weights was determined by bootstrapping with 5000 iterations. As shown in Table 3, all dimensions significantly contributed to their respective higher-order constructs. These findings validate the use of formative specification of the constructs, although from weight sizes, some dimensions have a stronger contribution than others.

The two dimensions have made substantial yet differential contributions in value co-creation. The outer weight of patient participation behavior is slightly higher than the outer weight of patient citizenship behavior. It signifies that those behaviors which are essential for the provision of healthcare services made a greater contribution towards the construct. Such behaviors include sharing information, collaboration, responsible participation, and interaction with healthcare providers. Patient citizenship behavior, which entails voluntary activities like feedback, helping others, advocating, and tolerance, made a considerable contribution to the construct, although relatively less so. However, the differential weights do not reflect a problem in the measurement of the construct because the formative dimensions are not supposed to contribute equally.

The discriminant validity of the constructs was tested using the Fornell–Larcker criterion and the Heterotrait–Monotrait ratio. The square root of the average variance extracted for each reflective construct was larger than its correlation with all other constructs, as shown in Table 4. The values for HTMT were also under the established cut-off point of 0.85 [44]. In order to conduct a more stringent test, the bootstrapping analysis was applied for assessing the HTMT values. None of the 95% confidence intervals had 1.00 as its upper boundary, which confirmed that the constructs were indeed empirically different. Special focus was paid to the constructs of patient empowerment, healthcare sustainability, and value co-creation due to their conceptual closeness.

Table 5 shows the discriminant validity.

Although patient empowerment and value co-creation both involve patient participation, their empirical distinction is consistent with their different conceptual roles. Patient empowerment represents the patient’s personal knowledge, confidence, and capacity to participate, whereas value co-creation represents interaction and joint contribution between patients and healthcare professionals. Healthcare sustainability concerns the continuity and dependability of the healthcare service environment rather than either individual capability or collaborative interaction.

### 4.3. Structural Model Assessment: Hypotheses Testing

Before analyzing the structural relationships, collinearity between the predictors was assessed by measuring the inner variance inflation factor values. The values of system quality, information quality, and service quality were found between 1.210 and 2.101, below the threshold value of 5.0. Specifically, system quality recorded a VIF of 1.210, information quality recorded 1.311, and service quality recorded 2.101. This shows that the relationships among the predictors were not collinear, and that their impact on the adoption of e-health could be estimated effectively [43].

The findings are as follows regarding hypothesis testing, which encompasses both direct and interaction hypotheses. Firstly, the hypothesis testing provided initial evidence for the direct effect (H1), indicating a significant relationship between system quality and the adoption of e-health (β = 0.312, t = 3.484, and *p* < 0.000). As a result, H1 was confirmed. Secondly, the second direct effect (H2), concerning the relationship between information quality and the adoption of e-health, was found to be positively significant (β = 0.202, t = 3.115, and *p* < 0.000). Therefore, H2 was also supported. Additionally, H3, which posited that service quality positively influenced the adoption of e-health, received support in the analysis (β = 0.274, t = 3.233, and *p* < 0.000). Hence, H3 was in line with our predictions. All of these results are detailed in Table 6.

Regarding the moderation predictions, this study incorporates two moderating variables: (i) Patient empowerment and healthcare sustainability, which includes hypotheses H4a to H4c. (ii) Patient empowerment value co-creation, encompassing hypotheses H5a to H5c. The results of the moderation analysis are discussed as follows:

The first interaction between system quality, patient empowerment, and healthcare sustainability on adopting e-health revealed a significant interaction effect (β = 0.068, t = 2.143, and *p* < 0.000), supporting H4a. The second interaction between information quality, patient empowerment, and healthcare sustainability on adopting e-health showed an insignificant interaction effect (β = 0.166, t = 2.239, and *p* < 0.000). Thus, H4b is accepted. The third interaction, examining the relationship between service quality and patient empowerment and healthcare sustainability on adopting e-health, exhibited a significant interaction effect (β = 0.289, t = 3.722, and *p* < 0.000), reinforcing H4c. The interaction between system quality and patient empowerment value co-creation concerning adopting e-health demonstrated a significant interaction effect (β = 0.215, t = 3.524, and *p* < 0.000), supporting H5a. However, H5b was insignificant (β = 0.106, t = 1.139, and *p* < 0.126). The last interaction, examining the relationship between service quality and patient empowerment value co-creation on adopting e-health, revealed a significant interaction effect (β = 0.348, t = 4.296, and *p* < 0.000), supporting H5c. All of the above findings are summarized in Table 7.

**Figure 2:**
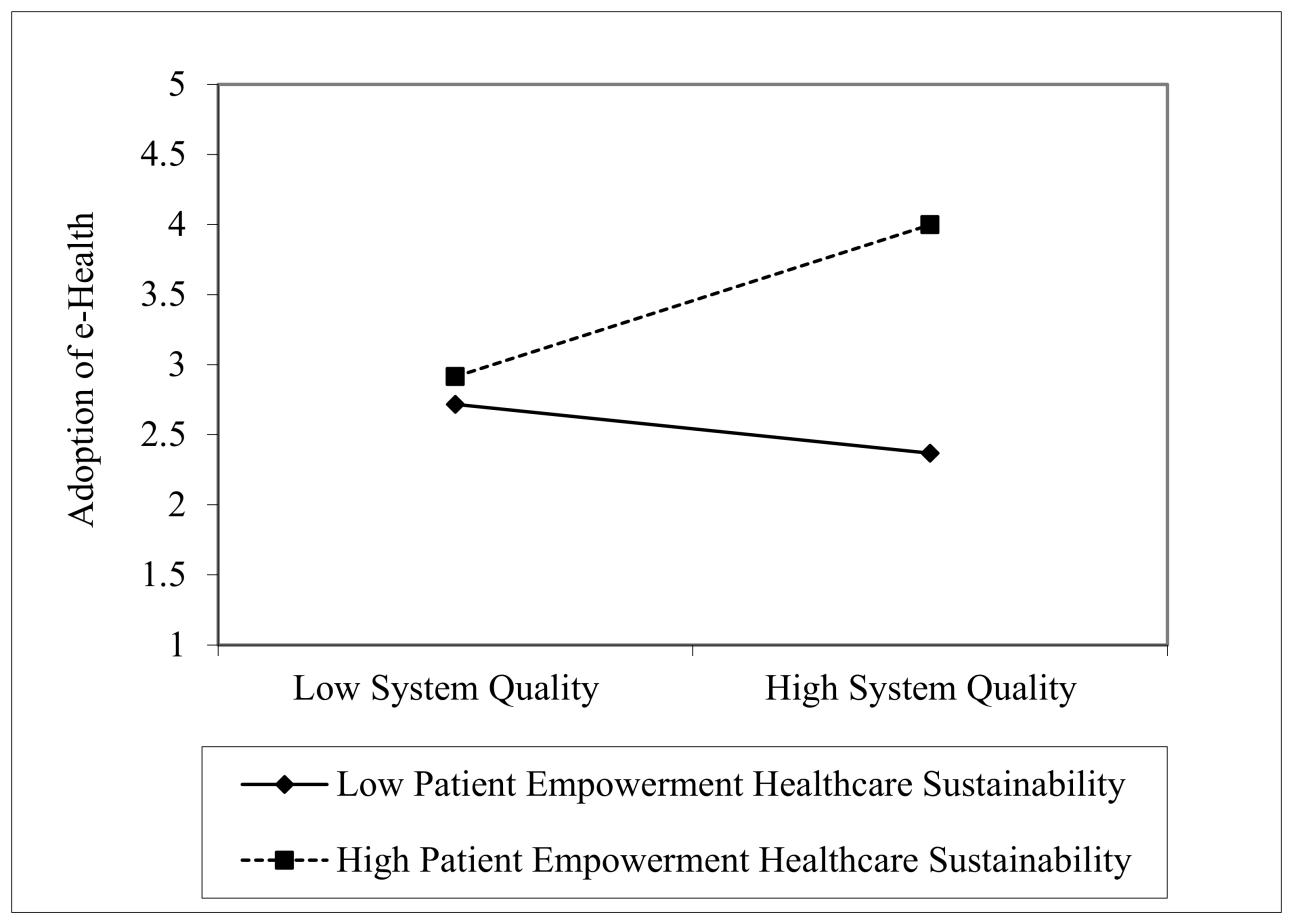
Interaction plot of system quality and patient empowerment healthcare sustainability on the adoption of e-health.

**Figure 3:**
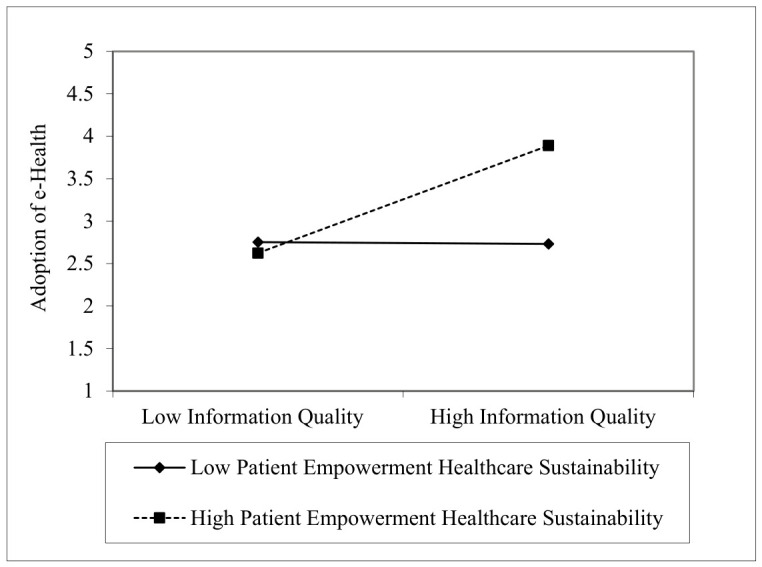
Interaction plot of information quality and patient empowerment healthcare sustainability on the adoption of e-health.

**Figure 4:**
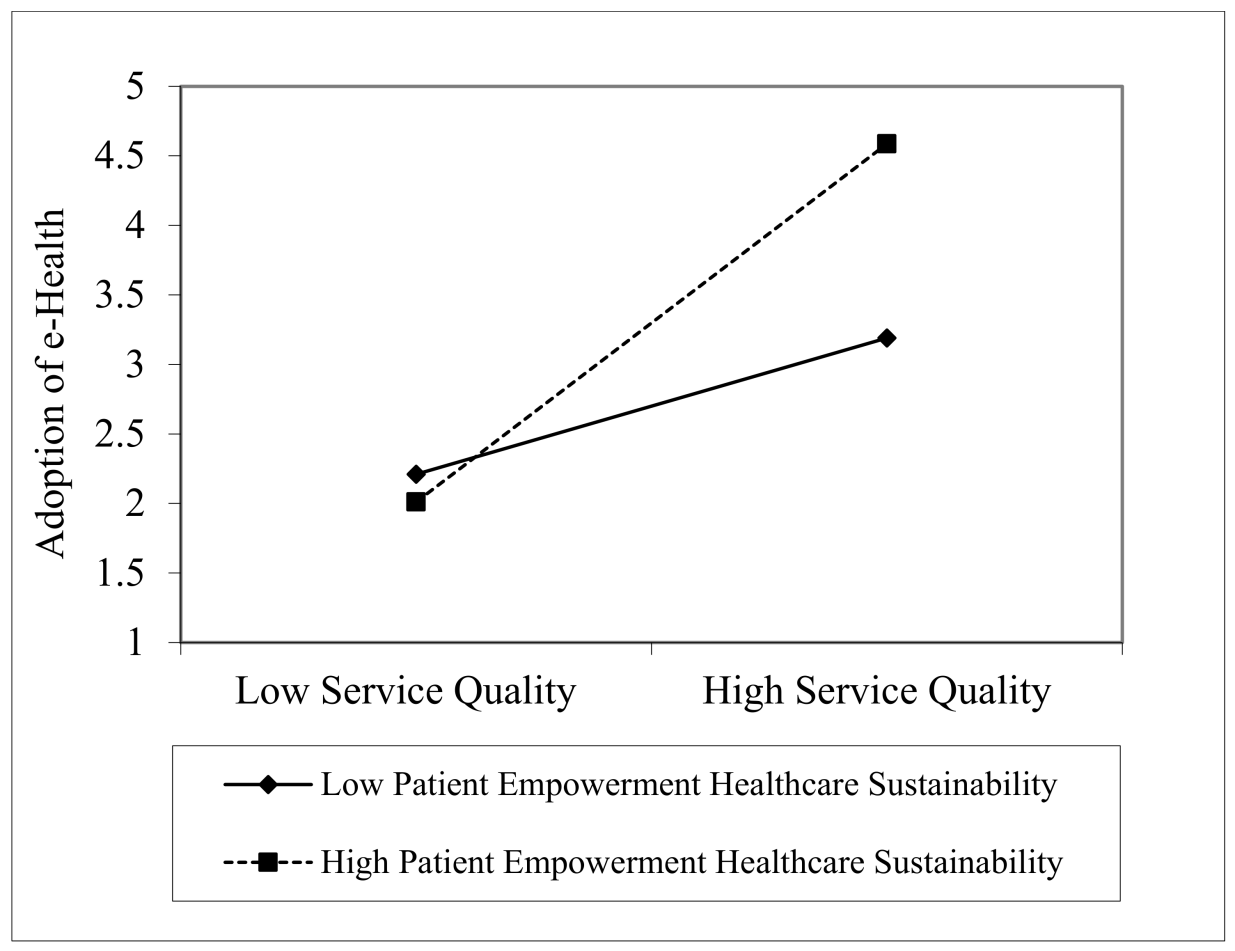
Interaction plot of service quality and patient empowerment on healthcare sustainability on the adoption of e-health.

**Figure 5:**
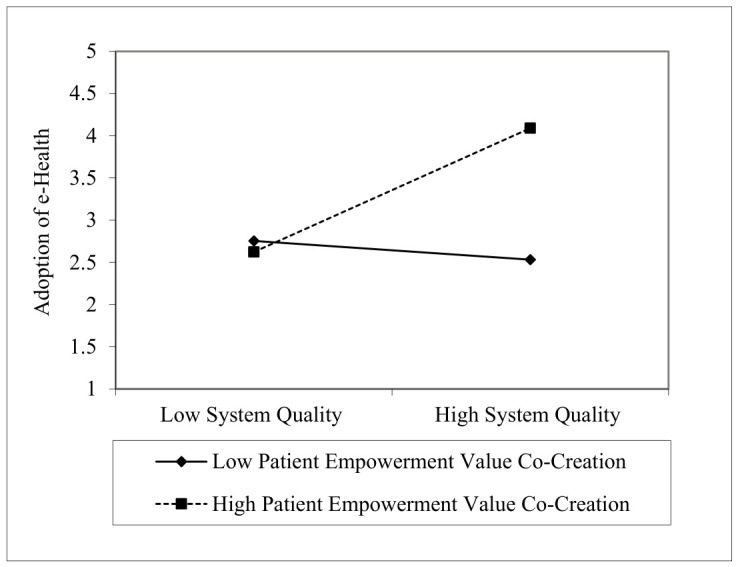
Interaction plot of system quality and patient empowerment value co-creation on the adoption of e-health.

**Figure 6:**
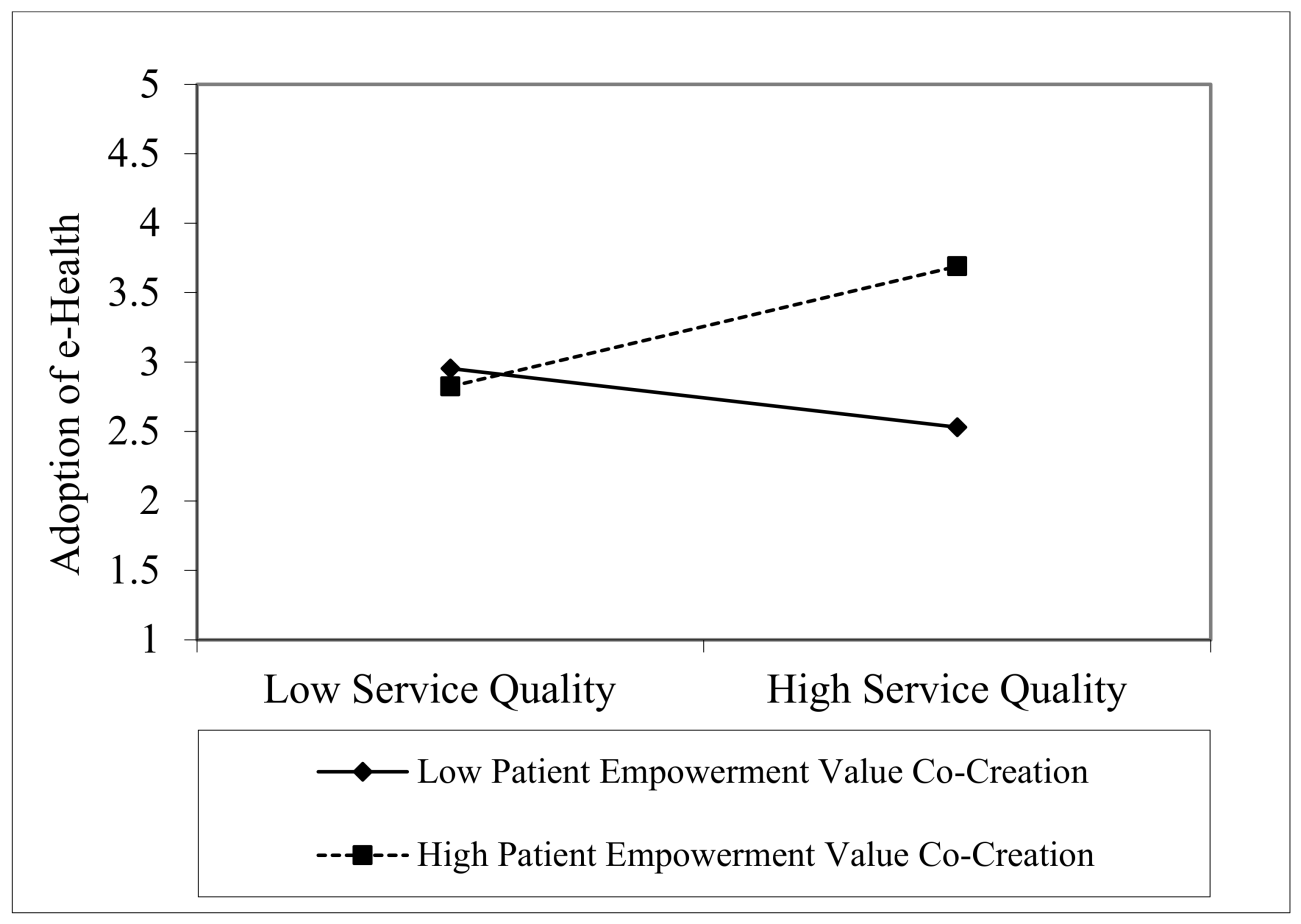
Interaction plot of service quality and patient empowerment value co-creation on the adoption of e-health.

According to Dawson [45], it is advisable to create interaction plots to better comprehend how interactions differ between high and low conditions. In our study, we utilized interaction plots for all the supported interactions to visualize the gradient of the slopes. Figure 2 illustrates that the positive relationship between system quality and the adoption of e-health is more pronounced when patient empowerment and healthcare sustainability are higher, as the slope is steeper. Figure 3 demonstrates that the positive relationship between information quality and the adoption of e-health becomes more prominent when patient empowerment healthcare sustainability is higher, showing a steeper slope. Figure 4 displays the interaction between service quality and patient empowerment healthcare sustainability on adopting e-health. It reveals that a high level of patient empowerment and healthcare sustainability enhances the relationship between service quality and adopting e-health. Figure 5 indicates that the relationship between system quality and the adoption of e-health is more pronounced when patient empowerment value co-creation is higher, as the slope is steeper. Finally, Figure 6 shows that the relationship between service quality and the adoption of e-health is strengthened by high levels of patient empowerment value co-creation, resulting in a steeper slope than at lower levels. Thus, these interaction plots provide a visual representation of how the relationships change under different conditions, offering valuable insights into moderating variables’ role in the study context.

## 5. Discussion

The findings indicate that system quality, information quality, and service quality have significant positive effects on patients’ adoption of e-health services. This is consistent with the Information Systems Success Model that states that users assess an information system through its technical performance, information outputs, and service support [1]. However, these three dimensions of quality did not have the same degree of influence. System quality exerted the strongest effect on e-health adoption, then on service quality, and lastly on information quality. The sequence can be explained by the role of technology in enabling users to adopt e-health services. While the benefits of information and service quality will be noticed after interacting with a platform, it will be impossible to do so if the platform is difficult to navigate, inaccessible, slow, insecure, or incompatible with patients’ devices. Therefore, system reliability, accessibility, usability, security, and response speed may be seen as necessary prerequisites to experiencing the other dimensions of quality [2,46]. The explanation is consistent with the Information Systems Success Model, where technical system performance influences users’ adoption of information systems [1,47]. In addition, this result supports the results of earlier e-health studies where the technical quality of digital healthcare platforms has been found to significantly affect users’ adoption and use [4,5].

Moreover, the results show that service quality had a considerable positive impact, which means that the adoption of e-health is not determined exclusively by technology. Patients need timely responses, dependable support, empathy, and assurance that healthcare and technical experts will resolve any problems they might encounter while using digital services. The importance of service quality is also explained by the healthcare context where responsive and reassuring interactions with patients are crucial during service delivery [48]. The result, thus, reflects the dual nature of e-health where digital technology should be complemented with responsive human and organizational services. Still, the lower impact of service quality compared to system quality might be due to the fact that patients are not able to experience responsive support if there are problems with navigating and using the platform. The finding is consistent with the literature, which indicates that service and technical quality jointly determine patients’ assessments of telehealth services [4].

Despite the weakest coefficient, the significant impact of information quality proves that the accuracy, relevance, completeness, timeliness, and understandability of information provided by a platform influence patients’ adoption decisions. Incorrect, incomplete, or outdated information can negatively impact users’ perception and, consequently, undermine patients’ ability to make informed healthcare decisions in digital healthcare [49]. The small coefficient should not be interpreted as the lack of importance of information quality. On the contrary, patients may see information quality as an inherent part of any authorized e-health service instead of the characteristic that distinguishes different e-health platforms. Information quality may also begin to influence patient decisions after overcoming initial difficulties with navigation and establishing trust in the platform. Technologies used to secure the authenticity and integrity of electronic health information may additionally increase patients’ trust in the service [50]. Thus, the results provide evidence for the sequential view: system quality enables users to interact with a platform, service quality ensures the quality of care, and information quality strengthens the usefulness and credibility of the service.

Collectively, these findings contribute to research by providing empirical support for the distinction between system quality, information quality, and service quality and their different impact on e-health adoption. Instead of seeing overall e-health quality as an undifferentiated concept, the results confirm the multidimensional nature of the Information Systems Success Model [1]. Healthcare organizations will be unable to achieve high levels of adoption if they offer only relevant information and responsive services, but the platform is not reliable from the technical point of view. Hence, to achieve sustainable adoption, it is necessary to pay attention to technical performance, informational credibility, and service responsiveness while realizing that system quality might be the basis for the effects of the other dimensions of quality [4,5].

The moderating impact of patient empowerment on the relationship between system quality and adoption of e-health platforms is facilitated by the health literacy of the patients, their digital self-efficacy, and motivation to get involved in their own care. Empowered patients not only receive e-health services, but have greater levels of confidence regarding their ability to find, process, and act on health information, interact with health professionals, and utilize digital technologies for managing their health. This means that when the system quality is sufficient, accessible, and easy to use, empowered patients are more capable of transforming those technical characteristics into actions related to their healthcare, including making appointments, monitoring their symptoms, viewing their medical records, and complying with prescriptions. Their digital self-efficacy also eliminates some of the uncertainties and concerns that people can experience while using new health technologies. Thus, patient empowerment positively influences the relationship between system quality and e-health adoption because more self-confident patients are more willing and capable of exploring the capabilities of the technologically advanced platform [13,51].

Patient empowerment can also increase the influence of information quality and service quality on patient behavior. People who are characterized by health literacy are better able to perceive the importance, precision, and usefulness of the information they are getting through the e-health platform. Such information is related to their health status and can assist them in making informed decisions about their health and treatments. At the same time, people with lower levels of health literacy will be less capable of understanding even high-quality information from a technical standpoint. Empowered patients are more likely to initiate interactions with healthcare professionals, ask questions, provide feedback, and seek clarification. This enables them to benefit more from responsive and personalized service support. The moderation effect works through the mechanism of active usage: patient empowerment makes patients more capable and motivated to transform system, information, and service quality into practical benefits instead of just noticing their presence [13,51].

Healthcare sustainability operates through a different mechanism. While patient empowerment concerns each patient’s abilities and initiative, healthcare sustainability involves the institutional factors that ensure the ongoing availability and efficiency of e-health services. A platform of sufficient system quality might be capable of encouraging adoption at first stages, but it can lose its influence when users start expecting disruptions, insufficient funding, lack of maintenance, or organizational support. A sustainable environment in healthcare can provide continuity through reliable digital infrastructure, qualified personnel, integration with the existing health services, and long-term commitment to telemedicine and remote monitoring. Such an environment reassures patients that the benefits provided by the system, information, and service quality are going to stay in place [52].

In this way, healthcare sustainability can strengthen the influence of the three quality dimensions by eliminating uncertainties about the ongoing use of e-health services. The stable institutional support helps to ensure the reliability of the system quality, continuous updating of health information, and provision of professional and technical help. Telemedicine and remote monitoring can also decrease the burden on physical healthcare facilities, make e-health services more available for geographically dispersed patients, and facilitate more efficient use of health resources [12,53]. Therefore, the moderation mechanisms of patient empowerment and healthcare sustainability are not interchangeable. Patient empowerment increases adoption by increasing the patient’s ability and desire to use the high-quality e-health services, while healthcare sustainability increases adoption by providing the institutional conditions that guarantee this use.

Further discussion is necessary in order to interpret the non-significant moderating effect of value co-creation on the connection between information quality and e-health adoption. First of all, while information quality includes the characteristics of the information provided through an e-health website, such as accuracy, completeness, relevance, timeliness, and clarity, they are mostly viewed by the patients as requirements to be met by any credible healthcare platform rather than benefits that become more important depending on the level of value co-creation. Therefore, once information meets such requirements, its impact on e-health adoption may occur regardless of the level of value co-creation.

Unlike information quality, the benefits of service quality and system quality depend on patient-provider collaboration, and patients can participate in improving system quality through giving feedback regarding the issues related to the usability, accessibility, functionality, and personalization of the platform. Patients can also help to improve the quality of services through dialog, joint decision-making, responsiveness, and adaptation to the needs of each patient individually. It can thus be concluded that those quality dimensions represent better opportunities for patients to interact with healthcare providers. In previous research, value co-creation was defined as a process of collaborative interaction between patients and professionals that helps to create knowledge and other resources in order to improve patients’ experiences with healthcare services and outcomes [15,32,52]. Information quality may be less directly shaped by collaborative effort but is a provider-controlled or system-based characteristic in cases where patients receive information without any active role in its generation and interpretation.

Moreover, the finding can be explained by considering the specific situation in Saudi Arabia, where digital health platforms are state-approved or authorized, and patients can be sure that the information is already verified and standardized. In this case, patient collaboration may add relatively little to the influence of the accuracy or timeliness of information on e-health adoption. Patients may still collaborate with the healthcare providers in order to give feedback, take part in making decisions about their treatment, etc., but these activities will be more related to service delivery and experience rather than to the quality of information provided by the platform. Digital access to health records and channels of communication with providers allows patients to participate in interactions but does not mean that they interpret and generate the information [29,30,31].

At the same time, there may also be a methodological explanation of the finding. The value co-creation measure may have assessed general collaboration, interaction with providers, and participation in the process but not necessarily joint production and interpretation of information provided through the platform. The non-significant interaction, thus, can be the result of the mismatch between the relational aspect included in the value co-creation measure and the information aspect of the information quality dimension. Finally, the cross-sectional design of the study may not allow detecting the changes that occur due to the patients’ participation in the collaborative process and change their ability to perceive information provided through the platform. Consequently, the result cannot be used as conclusive evidence that value co-creation does not moderate the relationship between information quality and e-health adoption. On the contrary, it means that, within the sample of the current study, there is no statistically detectable impact of value co-creation on the influence of information quality on e-health adoption.

Overall, this finding contributes to the extension of the Information Systems Success Model by demonstrating that contextual moderators do not necessarily operate uniformly across all quality dimensions. Value co-creation appears more likely to strengthen quality dimensions that patients can actively influence through interaction, feedback, and joint decision-making, particularly system quality and service quality. By contrast, information quality may be perceived as a relatively predetermined technical and professional standard whose effect on adoption is less dependent on collaborative participation. The finding therefore supports a selective rather than universal interpretation of the moderating role of value co-creation.

## 6. Implications

### 6.1. Knowledge Implications

The main objective of this study was to investigate the influence of IT quality attributes, namely system quality, information quality, and service quality, on the adoption of e-health services. This study extends the existing literature by introducing patient empowerment, healthcare sustainability, and value co-creation as distinct moderating variables in the relationships between IT quality attributes and e-health adoption. By integrating these contextual factors into the Information Systems Success Model, the study provides a more comprehensive understanding of the conditions under which IT quality attributes translate into successful e-health adoption.

Situated at the intersection of IT quality attributes, patient empowerment, healthcare sustainability, and value co-creation, this research highlights the importance of both technological and contextual factors in shaping healthcare outcomes. The findings suggest that the effective utilization of high-quality information systems can enhance patient engagement and support individuals in taking a more active role in their healthcare decisions. Patient empowerment plays a key role in encouraging informed decision-making and self-management, while healthcare sustainability ensures that such engagement can be maintained over time through efficient and accessible healthcare delivery systems.

From a practical perspective, these findings suggest that policymakers and healthcare providers should not only focus on improving system, information, and service quality, but also foster patient engagement and collaborative healthcare practices to maximize the effectiveness of e-health systems. In essence, this study underscores the critical roles of IT quality, patient empowerment, healthcare sustainability, and value co-creation in advancing a more sustainable and patient-centered healthcare system.

### 6.2. Practical Implications

The study provides several practical implications for healthcare organizations, e-health developers, and policymakers. Since system quality proved to have the most influence on e-health adoption, the organizations in question should pay attention to the technical reliability and usability of the digital health platforms. To that end, the high level of system availability and low levels of system loading and response time need to be ensured. Moreover, compatibility with various mobile devices and operating systems, as well as simplicity of navigation regardless of the digital experience level of the users, are crucial aspects in this regard. In addition, it is essential to carry out the testing of platforms’ usability in cooperation with patients and not just developers and healthcare professionals [2,4,46].

The significant impact of information and service qualities implies that technical improvement needs to be complemented by information governance and provision of high-quality services in terms of response to patient inquiries, resolution of any issues that occur, and reporting and explanation of test results or other information provided by healthcare professionals. Help desks, chatbots, escalation procedures, and digital health support staff may decrease patients’ frustration and provide reassurance regarding the availability of the necessary assistance. It is important to train the healthcare professionals in digital communication in order to ensure empathy, responsiveness, and clarity in their interactions with the patients.

Patient empowerment should be regarded as an aspect of e-health implementation and not as its automatic result. There are several means that can be employed by the organizations in order to empower patients and make them more capable of using digital health services. These include orientation sessions, short instructional videos, demonstrations, FAQs, and assistance for less digitally literate patients. Patient empowerment can also be achieved by incorporating the health literacy function into digital health services to allow patients to comprehend the information about their health conditions and take action. Symptom trackers, medication reminders, access to medical records, and individual health goals may increase patients’ involvement in their health management. However, these tools should be combined with the advice from healthcare professionals since mere provision of information is insufficient in this case [13,51].

The results related to the impact of value co-creation imply that it is critical to involve patients in the design and improvement of e-health services. The organizations in question may establish patient advisory boards, regularly conduct user experience surveys, and provide obvious feedback channels where patients can complain about usability issues or suggest features that may be added to platforms. In addition, patients may be involved in the testing of the updates before their implementation. On the clinical level, value co-creation can be stimulated through shared decision-making, creation of individual care plans, bidirectional communication, and opportunities for patients to discuss how digital services can help them maintain their health. All of these measures will increase the effects of the system and service quality on e-health adoption because of patients’ satisfaction with their experience and preferences [15,32,52].

The sustainable development of healthcare requires long-term commitments on behalf of organizations and policymakers. Healthcare providers should avoid treating digital health platforms as temporary projects and continue devoting resources to their maintenance, updating of software, cybersecurity, staff development, and technical support. Digital health services also should be integrated into the current clinical workflow and patients’ records to prevent the separation of online and face-to-face healthcare. The sustainability of digital health services may be supported by the policies related to the development of common interoperability and data protection standards, coordination of efforts among healthcare institutions and evaluation of the accessibility and performance of these services. In addition, policies need to address the issue of inequality in digital access through support of patients living in rural areas, elderly people, and those having problems with digital literacy and connection to the Internet. Otherwise, the improvements in system, information, and service quality will be experienced by the patients having all the necessary resources to use digital health technologies.

Finally, healthcare organizations should track the adoption of e-health services in terms of performance indicators and not merely the number of registered users. The appropriate indicators may include the rate of active use, frequency of repeated use, completion rate of appointments, response time, technical complaints, patient satisfaction, accessibility outcomes, and discontinuation rates. Furthermore, segmenting these indicators by age, location, digital experience, and health state will allow for identifying particular groups having problems with the platform usage.

## 7. Limitations and Directions for Future Research

Although this study provides valuable insights, several limitations should be acknowledged. First, the findings are context-specific to Saudi Arabia, and cultural, social, and healthcare system characteristics may influence the observed relationships. As such, the generalizability of the results to other regions should be approached with caution. Secondly, the study employed a cross-sectional design, which limits the ability to establish causal relationships among the variables over time. Future research could adopt longitudinal approaches to better capture changes in e-health adoption behavior. Third, the use of purposive sampling may limit the representativeness of the sample and restrict the generalizability of the findings to the broader patient population. In addition, the reliance on self-reported survey data may introduce response bias, despite the procedural steps taken to minimize such effects.

Future research can build on this study by incorporating more diverse samples across different regions and healthcare systems to enhance external validity. Expanding the model to include additional variables, such as socioeconomic factors, digital health literacy, and healthcare provider characteristics, may provide a more comprehensive understanding of e-health adoption dynamics.

Furthermore, qualitative or mixed-method approaches could offer deeper insights into contextual and behavioral factors, including patient experiences and cultural influences that are not easily captured through quantitative methods. Future studies may also examine the impact of ongoing technological advancements and policy initiatives on e-health adoption, particularly within rapidly evolving healthcare environments such as Saudi Arabia.

## 8. Conclusions

This study examined the determinants of e-health adoption in Saudi Arabia, focusing on the roles of system quality, information quality, and service quality, and how patient empowerment, healthcare sustainability, and value co-creation moderate these relationships. The findings highlight that all three quality dimensions significantly influence e-health adoption, with patient empowerment enhancing certain adoption pathways.

These results provide empirical insights into how e-health implementation can be supported through IT quality improvements and patient-centered strategies. The study offers practical guidance for policymakers and healthcare providers seeking to enhance e-health adoption in alignment with Saudi Vision 2030. Future research may build on these findings by employing longitudinal designs, broader samples, and additional contextual variables.

## Figures and Tables

**Table 1:** Common method variance assessment via full collinearity estimate criteria.

Variable	System Quality	Information Quality	Service Quality	Patient Empowerment	Healthcare Sustainability	Patient Empowerment Value Co-Creation
VIF	1.210	1.311	2.101	1.245	1.198	1.137

Note(s): VIF = variance inflation factor.

**Table 2:** Measurement model: Item loading/weight, construct reliability and convergent validity.

1st-Order Constructs	2nd-Order Constructs		Items	Scale	Loading/Weight	CR/VIF	AVE/t-Value	*p*-Value
System Quality			SQ-1	Reflective	0.772	0.753	0.641	n.a.
			SQ-2		0.755			
			SQ-3		0.800			
			SQ-4		0.795			
Information Quality			IQ-1	Reflective	0.785	0.818	0.711	n.a.
			IQ-2		0.861			
			IQ-3		0.855			
			IQ-4		0.782			
Service Quality			SQ-1	Reflective	0.853	0.813	0.689	n.a.
			SQ-2		0.770			
			SQ-3		0.810			
			SQ-4		0.771			
			SQ-5		0.814			
Health Literacy			HL-1	Reflective	0.807	0.836	0.715	n.a.
			HL-2		0.755			
			HL-3		0.838			
			HL-4		0.831			
Patient Participation			PP-1	Reflective	0.819	0.831	0.668	n.a.
			PP-2		0.874			
			PP-3		0.842			
Patient Control			PC-1	Reflective	0.785	0.833	0.571	n.a.
			PC-2		0.863			
			PC-3		0.879			
			PC-4		0.755			
Communication with Healthcare Professionals			CHP-1	Reflective	0.761	0.744	0.615	n.a.
			CHP-2		0.860			
			CHP-3		0.832			
			CHP-4		0.810			
			CHP-5		0.746			
			CHP-6		0.778			
			CHP-7		0.819			
			CHP-8		0.859			
			CHP-9		0.807			
			CHP-10		0.814			
	Patient Empowerment	Healthcare Sustainability	Health Literacy	Formative	0.334	1.461	3.491	0.000
			Patient Participation		0.322	1.486	3.896	0.002
			Patient Control		0.321	1.264	4.200	0.000
			Communication with Healthcare Professionals		0.320	2.391	2.175	0.001
Patient Participation Behavior			PPB-1	Reflective	0.749	0.831	0.671	n.a.
			PPB-2		0.844			
			PPB-3		0.743			
			PPB-4		0.841			
			PPB-5		0.844			
			PPB-6		0.745			
			PPB-7		0.850			
			PPB-8		0.787			
			PPB-9		0.849			
			PPB-10		0.853			
Patient Citizenship Behavior			PCB-1	Reflective	0.782	0.781	0.625	n.a.
			PCB-2		0.881			
			PCB-3		0.756			
			PCB-4		0.811			
			PCB-5		0.786			
	Value Co-Creation		Patient Participation Behavior	Formative	0.441	1.387	3.574	0.000
			Patient Citizenship Behavior		0.353	1.364	3.645	0.000
Adoption of e-Health			AEH-1	Reflective	0.799	0.871	0.682	n.a.
			AEH-2		0.891			
			AEH-3		0.787			
			AEH-4		0.783			
			AEH-5		0.847			
			AEH-6		0.855			
			AEH-7		0.760			
			AEH-8		0.810			

Note(s): CR = composite reliability, VIF = variance inflation factor, AVE = average variance extracted, n.a = not applicable.

**Table 3:** Assessment of the formative higher-order constructs.

Higher-Order Construct	First-Order Dimension	Outer Weight	VIF	t-Value	*p*-Value	Assessment
Patient Empowerment	Health Literacy	0.334	1.461	3.491	<0.001	Significant
Patient Empowerment	Patient Participation	0.322	1.486	3.896	0.002	Significant
Patient Empowerment	Patient Control	0.321	1.264	4.200	<0.001	Significant
Patient Empowerment	Communication with Healthcare Professionals	0.320	2.391	2.175	0.001	Significant
Value Co-Creation	Patient Participation Behavior	0.441	1.387	3.574	<0.001	Significant
Value Co-Creation	Patient Citizenship Behavior	0.353	1.364	3.645	<0.001	Significant

Note: VIF = variance inflation factor. Significance was assessed using bootstrapping with 5000 resamples.

**Table 4:** Descriptive statistics, correlation matrix, and discriminant validity via Fornell and Larcker.

Constructs	Mean	SD	1	2	3	4	5	6
1. System Quality	3.647	0.585	**0.778**					
2. Information Quality	3.472	0.551	0.602	**0.713**				
3. Service Quality	4.184	0.637	0.432	0.514	**0.829**			
4. Patient Empowerment Healthcare Sustainability	4.513	0.519	0.134	0.165	0.483	**0.809**		
5. Patient Empowerment Value Co-Creation	4.211	0.549	0.335	0.583	0.472	0.305	**0.742**	
6. Adoption of e-Health	4.312	0.521	0.408	0.574	0.555	0.248	0.602	**0.717**

Note(s): SD = Standard Deviation. Bold values on the diagonal in the correlation matrix are square roots of AVE (variance shared between the constructs and their respective measures). Off-diagonal elements below the diagonal are correlations among the constructs, where values between 0.13 and 0.16 are significant at *p* < 0.05, and values above 0.16 are significant at *p* < 0.01 (two-tailed test).

**Table 5:** Discriminant validity via HTMT.

Constructs	1	2	3	4	5	6
1. System Quality						
2. Information Quality	0.454					
3. Service Quality	0.399	0.679				
4. Patient Empowerment Healthcare Sustainability	0.527	0.604	0.529			
5. Patient Empowerment Value Co-Creation	0.189	0.264	0.359	0.558		
6. Adoption of e-Health	0.784	0.633	0.667	0.593	0.199	

Note(s): HTMT should be lower than 0.85.

**Table 6:** Structural path analysis: direct effect.

						Bias and Corrected Bootstrap 95% CI		
Hypothesis	Relationship	Std Beta	Std Error	t-Value	*p*-Value	BCI 95% LL	BCI 95% UL	Decision
H-1	System Quality -> Adoption of e-Health	0.312	0.088	3.484	0.000	0.125	0.289	Supported
H-3	Information Quality -> Adoption of e-Health	0.202	0.054	3.115	0.000	0.313	0.553	Supported
H-3	Service Quality -> Adoption of e-Health	0.274	0.062	3.233	0.000	0.281	0.381	Supported

Note(s): n = 377. Bootstrap sample size = 5000. LL = lower limit; CI = confidence interval; UL = upper limit; 95% bias-corrected CI.

**Table 7:** Structural path analysis: The interaction effect.

	(1) Patient Empowerment Healthcare Sustainability					Bias and Corrected Bootstrap 95% CI		
Hypothesis	Relationship	Std Beta	Std Error	t-Value	*p*-Values	BCI 95% LL	BCI 95% UL	Decision
H-4a	System Quality × Patient Empowerment Healthcare Sustainability --> Adoption of e-Health	0.068	0.027	2.143	0.000	0.023	0.123	Supported
H-4b	Information Quality × Patient Empowerment Healthcare Sustainability --> Adoption of e-Health	0.166	0.096	2.239	0.000	0.010	0.274	Supported
H-4c	Service Quality × Patient Empowerment Healthcare Sustainability --> Adoption of e-Health	0.289	0.088	3.722	0.000	0.020	0.076	Supported
	**(2) Patient Empowerment Values Co-Creation**							
H-5a	System Quality × Patient Empowerment Value Co-Creation --> Adoption of e-Health	0.215	0.061	3.524	0.000	0.124	0.321	Supported
H-5b	Information Quality × Patient Empowerment Value Co-Creation --> Adoption of e-Health	0.106	0.093	1.139	0.126	0.010	−0.274	Not Supported
H-5c	Service Quality × Patient Empowerment Value Co-Creation --> Adoption of e-Health	0.348	0.081	4.296	0.000	0.237	0.485	Supported

Note(s): n = 377. Bootstrap sample size = 5000. LL = lower limit; CI = confidence interval; UL = upper limit; 95% bias-corrected CI.

## Data Availability

Data will be made available on reasonable request.
